# Treatment Comparison in Rheumatoid Arthritis: Head-to-Head Trials and Innovative Study Designs

**DOI:** 10.1155/2014/831603

**Published:** 2014-04-16

**Authors:** Ennio Giulio Favalli, Serena Bugatti, Martina Biggioggero, Roberto Caporali

**Affiliations:** ^1^Department of Rheumatology, Gaetano Pini Institute Via Gaetano Pini 9, 20121 Milano, Italy; ^2^Division of Rheumatology, University of Pavia, IRCCS Policlinico San Matteo Foundation, Piazzale Golgi 2, 27100 Pavia, Italy

## Abstract

Over the last decades, the increasing knowledge in the area of rheumatoid arthritis has progressively expanded the arsenal of available drugs, especially with the introduction of novel targeted therapies such as biological disease modifying antirheumatic drugs (DMARDs). In this situation, rheumatologists are offered a wide range of treatment options, but on the other side the need for comparisons between available drugs becomes more and more crucial in order to better define the strategies for the choice and the optimal sequencing. Indirect comparisons or meta-analyses of data coming from different randomised controlled trials (RCTs) are not immune to conceptual and technical challenges and often provide inconsistent results. In this review we examine some of the possible evolutions of traditional RCTs, such as the inclusion of active comparators, aimed at individualising treatments in real-life conditions. Although head-to-head RCTs may be considered the best tool to directly compare the efficacy and safety of two different DMARDs, surprisingly only 20 studies with such design have been published in the last 25 years. Given the recent advent of the first RCTs truly comparing biological DMARDs, we also review the state of the art of head-to-head trials in RA.

## 1. Introduction

Based on the wrong assumption of a possible interference with proliferation of connective tissues, methotrexate (MTX) was first trialled for the treatment of rheumatoid arthritis (RA) in 1962 [1]. Definite approval of MTX as a therapy for active RA came in 1988 after two placebo-controlled studies involving a total of 224 patients treated for a maximum of 24 weeks [2, 3]. Much has changed since then in drug discovery and trial design in RA. The identification of tumor necrosis factor (TNF) as a key player in the inflammatory and destructive pathways of the disease initiated a landmark shift of interest away from agents with poorly understood mechanisms of action towards therapies targeted to key molecules and cells involved in RA pathogenesis [4]. Advances in understanding of the role of T cells, B cells, and cytokines such as IL-6 have paved the way to the development of additional biological drugs beyond TNF-inhibitors, such as abatacept (ABT), rituximab, and tocilizumab (TCZ) [5–10]. These have come to formal approval after randomised controlled trials (RCTs) mostly adherent to the recommendations from the US Food and Drug Administration (FDA) and the European Agency for the Evaluation of Medicinal Products (EMA). Specific requirements include long-term RCTs (12 to 24 months in duration) evaluating radiographic progression, and patient-reported physical function in addition to accepted outcomes assessing signs and symptoms [11, 12].  Table 1 summarises RCTs of biological disease-modifying antirheumatic drugs (DMARDs) that have supported regulatory labelling [13–45]. After more than ten years of experience, biological DMARDs have consistently shown good efficacy and safety in patients with RA [46–51].

The ever-increasing plethora of effective treatment options for patients with RA undoubtedly reflects the vitality of research in this area. The paradox, however, is that rheumatologists have little or no idea of how to approach an individual patient to best utilise this vast arsenal. As a proof, updated recommendations for the management of RA still refer to homogeneous disease populations (with the exception of few and ill-defined prognostic factors) and, most importantly, do not assist in the choice and optimal sequencing of available biological DMARDs [52–54]. This area of uncertainty is likely to increase with the upcoming introduction of biosimilar and targeted synthetic DMARDs on the market.

Classic RCTs sponsored by pharma industries to assess the efficacy and safety of new compounds clearly do not fit well with the urgent need of improving decisions that affect medical care at the levels of both policy and the individual. Most of the RCTs in RA indeed exclude commonly used comparator interventions and clinically relevant patient subgroups. These exclusions diminish the ability to understand the relative merits of different interventions and the generalisability of the trial results [55]. Although comparative efficacy and effectiveness can be informed by analysis of observational data, decision modelling, and other tools (reviewed in [56, 57]), the RCT still remains the most rigorous method for comparing interventions. However, trial designs should be substantially rethought in order to allow reliable assumptions on the effectiveness of different interventions among patients in typical day-to-day practice. In this review, we will first briefly summarise how and to what extent the dearth of evidence from comparative RCTs in RA can be partially counterweighted by indirect comparisons. We will then examine some of the possible implementations to classic RCTs, such as the inclusion of active comparators and new trial designs, to align research methods to current demands. In light of the recent advent of the first RCTs truly comparing biological DMARDs, we will also review the state of the art of head-to-head trials in RA.

## 2. Indirect Comparisons and Meta-Analysis

In contrast to direct within-trial comparison, in indirect comparisons, the effects of interventions are compared to each other by their performance against a common comparator. Quantitative results of several similar studies comparing the same intervention with the same comparator can be combined by means of meta-analysis to summarise the available evidence into a pooled estimate of the outcome of interest (pairwise meta-analysis). Furthermore, multiple different pairwise comparisons across a range of different interventions can be combined into network meta-analysis (also known as mixed treatment comparisons or multiple treatment meta-analyses) [58, 59].

Of key importance in indirect comparisons is not to break randomisation, thus preserving the advantages of RCTs. If one trial compares drug A versus placebo and a second trial compares drug B versus placebo, it is incorrect to simply compare the absolute efficacy observed with drug A with that observed with drug B. Indeed, part of the absolute efficacy can be attributed to the drug, whereas another part is due to a placebo effect. Furthermore, differences in absolute treatment effects may be a result of different baseline prognostic factors. In order not to break randomisation, one can only compare the relative effect of drug A versus placebo from one trial with the relative effect from other trials (adjusted indirect comparison) [60].

Basic assumptions underlying indirect comparisons include that results from different trials should be sufficiently homogeneous [61] by either fixed-effects models or random-effects models. In fixed-effects models, it is assumed that differences in true relative treatment effects are only caused by the difference in treatment and no other factors. In random-effects models, differences in study-specific treatment effects (beyond the differences attributable to the interventions compared) are exchangeable, and heterogeneity is constant between the different comparisons [62]. Another assumption for an adjusted indirect comparison to be valid is similarity [61]. This means that patients included should be sufficiently similar in the two sets of placebo-controlled trials, so that the relative effect estimated by trials of A versus C is generalisable to patients in trials of B versus C, and the relative effect estimated by trials of B versus C is generalisable to patients in trials of A versus C. Last, when both direct and indirect evidence is available, an assumption of consistency is required to quantitatively combine the direct and indirect estimates [61]. Possible causes of discrepancy (inconsistency) between the direct and indirect evidence include the play of chance, invalid indirect comparison, bias in head-to-head comparative trials, and clinically meaningful heterogeneity across trials.

Conclusions from meta-analysis are drawn by applying a statistical inference technique, which can be either frequentist or Bayesian [63]. With a frequentist approach, the result of the meta-analysis is a point estimate along with a 95% confidence interval. Bayesian methods involve a formal combination of a priori probability distribution (that reflects a priori belief of the possible values of the pooled effect) with a likelihood distribution of the pooled effect based on the observed data to obtain a posterior probability distribution of the pooled effect. The likelihood informs us about the extent to which different values for the parameter of interest is supported by the data. As such, the posterior distribution obtained with the Bayesian approach can be interpreted in terms of probabilities, which allows for a more intuitive interpretation of the results.

Although being increasingly adopted to compare the effects of different treatments in many medical areas, indirect comparisons are not immune to conceptual and technical challenges. As treatments being compared have not been randomised directly within the individual trials, standard meta-analysis provides evidence of an observational nature and thus suffers from the limitations of observational studies [61]. Furthermore, sometimes inconsistency cannot be explained after considering effect modifiers. A recent meta-epidemiological study indeed identified 14% of inconsistency between direct and indirect comparisons [64].

Surprisingly, evidence for conventional DMARDs (MTX, leflunomide [LEF], and sulfasalazine [SSZ]) in RA coming from network meta-analysis combining direct and indirect comparisons does not support the superiority of one DMARD over another [65]. Limitations may stem from the wide differences in MTX dosing across different trials. The preferred use of MTX in most patients versus other oral DMARDs is thus rather supported by extensive clinical experience over the years [66]. For biological DMARDs, as head-to-head comparisons are only exceptions, thus, network meta-analysis is the sole informative tool for comparative effectiveness. Disappointingly, many of the published comparisons lead to different conclusions. As an example, a 2009 Cochrane overview failed to recognise significant differences in efficacy among the available biological DMARDs (with the exception of anakinra), whilst the safety profile favoured etanercept (ETN) [67]. In contrast, Schmitz and colleagues [68] reported superiority of ETN compared with infliximab (IFL) and golimumab and of certolizumab compared to infliximab and adalimumab (ADA). A number of factors may account for such inconsistency, including the RCTs being considered, the analysis of potential sources of heterogeneity, and the efficacy outcomes assessed [69]. Additionally, some confounders, such as period of enrolment of different RCTs, cannot be adequately corrected, limiting the possibility of indirect comparisons for specific outcomes (e.g., radiographic progression) [70]. These shortcomings currently hamper the use of available indirect comparisons as part of formal decision making strategies in RA.

## 3. Randomised Controlled Trials: New Designs to Improve Comparative Effectiveness 

The classic RCT that most rheumatologists are familiar with is the two-armed, parallel-group efficacy trial comparing the experimental treatment with placebo. This type of RCT is clearly not aligned with the need of determining the optimal strategy for individual patients in a community-based setting. The use of active comparators instead of placebo will be discussed in a further section. Here, we will summarise possible trial implementations aimed at individualising treatments in real-life conditions.

### 3.1. Implementation by Study Design

#### 3.1.1. N-of-1 Trials

The development of n-of-1 or single subject clinical trials is based on the recognition of the tremendous heterogeneity of diseased populations and the need of individualised treatments. In a n-of-1 trial, the individual patient is considered as the sole unit of observation, with the ultimate goal of determining the optimal or best intervention for that specific patient using objective data-driven criteria [71]. Although n-of-1 trials, by definition, eschew consideration of the population-level effects of an intervention, combining and evaluating multiple n-of-1 trials through meta-analysis can allow generalisability of the results [72]. The typical design of a n-of-1 trial is a within patient randomised, double-blind, and crossover trial. The unit of randomisation is the treatment sequence for an individual patient, and a treatment cycle includes an exposure to each therapy. In contrast to classic randomised crossover trials, where the individual is randomised to one group or another, in n-of-1 trials each participant receives each intervention at different time frames of the study.

N-of-1 trials have not been frequently adopted in rheumatology, with the exception of studies of pain medications in osteoarthritis. A study by Yelland et al. [73] provides a good example. A comparison of celecoxib and paracetamol was assessed. The design of the trial was based on a double-blind, crossover comparison where a subject took either celecoxib or sustained-release paracetamol for three pairs of 2-week periods. The order of the drugs during each pairing was random. Both patients and physicians did not know the order of the drug regimens until after the study was completed. Statistical analyses were conducted using Bayesian methods. The aggregate results showed that most (80%) patients completing the trial had a similar response to celecoxib as to paracetamol.

#### 3.1.2. Cluster Randomised Trials

In cluster RCTs, randomisation is by group (such as communities, families, or medical practices) rather by individual patient. Advantages of cluster RCTs over individually randomised controlled trials include the ability to study interventions that cannot be directed toward selected individuals and the ability to control for “contamination” across individuals, that is, the unintentional spillover of intervention effects from one treatment group to another. However, because of the dependence (or clustering) between individual units sample, cluster RCTs require more participants to obtain the same statistical power and are more complex to design, execute, and analyse [74].

Cluster RCTs are becoming increasingly common in health services research, being particularly appropriate for evaluating interventions aimed at changing behaviour in patients or practitioners or changing organisation of services. In RA, the effectiveness of systematic monitoring of disease activity in daily practice was confirmed in a multicentre cluster RCTs published in 2005 [75]. Twenty-four rheumatology outpatient centres were randomly allocated to systematic monitoring (0–4–12–24 weeks) using 28 joints disease activity score (DAS28) versus usual care. At 24 weeks, low disease activity (DAS28 ≤ 3.2) was achieved by 31% of the patients in the DAS28 group compared to 16% of the patients receiving usual care (*P* = 0.028) due to prompt changes in DMARD treatment.

### 3.2. Implementation by Outcome of Interest

#### 3.2.1. Pragmatic Trials

In efficacy (or explanatory) RCTs, extended inclusion and exclusion criteria are used to identify a clearly defined population of participants who would benefit from the intervention under investigation. Although efficacy trials, if correctly designed and executed, lead to statistically credible results, the applicability of these results to real-life practice may be questionable. Indeed, the same characteristics that account for the high internal validity (well-defined inclusion and exclusion criteria, blinding, and controlled environment) can hamper external validity, that is, the ability to generalise the results in an extended population and clinical setting. Pragmatic RCTs, on the other hand, are designed to test interventions in the full spectrum of everyday clinical practice in order to maximise applicability and generalisability [76]. Common elements of such trials include clinically effective comparators, study patients with common comorbid conditions and diverse demographic characteristics, and providers from community settings. Primary and secondary outcomes are patient-centered. The distinction between an explanatory and a pragmatic trial in real life is not that easy. The Pragmatic-Explanatory Continuum Indicator Summary (PRECIS) provides a useful framework to help researchers design pragmatic trials [77]. This tool identifies important domains (such as eligibility criteria, flexibility of the intervention, patient adherence, practitioner expertise, follow-up intensity, and outcomes) that should be considered during protocol development of pragmatic RCTs. Pragmatic trials arguably combine the advantages of randomisation (high internal validity) and observational research (high external validity). However, they also have important shortcomings. The increased variance due to the inclusion of chronic/poorly responsive/comorbid patients, insensitive or problematic outcome parameters, and inadequate sample size increases the risk of a *β*-error (failure to detect a difference although there is one), and unblinded designs can induce different kinds of biases.

In RA, the most cited example of a pragmatic trial is the Dutch Behandel Strategieen (BeSt) study [78]. Patients with early, untreated, and active RA were randomly allocated to 1 of 4 treatment groups. Treatment strategies included sequential monotherapy (group 1), step-up combination therapy (group 2), initial combination therapy with tapered high-dose prednisone (group 3), or initial combination therapy with IFL (group 4). Treatment adjustments were made on the basis of the treat-to-target and tight control principles. Primary endpoints were functional ability and radiographic joint damage. Despite several limitations, such as unblinding and intention-to-treat analysis, the BeSt study has contributed to significant advances in the management of RA by demonstrating that, in the majority of patients, a goal-steered, dynamic treatment towards tight control of disease activity ensures good clinical and radiographic outcomes irrespective of the type, combination, and sequencing of therapies [79].

#### 3.2.2. Adaptive Trials

A conventional study is planned using assumptions about critical elements of the study design, such as population means or event rates, variance, dose-response effect size, discontinuation rates, that are not precisely known but are only estimated. When the prestudy estimates are inaccurate, a conventional study may fail to achieve its goal. Data accumulating during the course of the study, however, could provide improved knowledge of relevant parameters if those data could be examined. Adaptive RCTs are designed to change or adapt in response to information generated during the trial [80, 81]. This could make the studies more efficient (e.g., shorter duration, fewer patients), more likely to demonstrate an effect of the drug if one exists, or more informative. Importantly, adaptations (or changes) should not be ad hoc, but by design, based on prospectively planned, prespecified analyses of interim data. Points of weakness of adaptive designs include feasibility, validity, integrity, efficiency, and flexibility [81, 82].

Of the various adaptive design trials [81], biomarker-driven adaptive studies perhaps offer the most attractive prospects. Predictive biomarkers can be selected from a wide array of prognostic biomarkers (which are useful for projecting the natural history of a disease independent of therapy) to define a specific subgroup of patients for which treatment will be beneficial. An example of a predictive marker is the presence or absence of K-Ras mutations in colorectal cancers; patients without K-Ras mutations benefit from antiepidermal growth factor receptor therapy, whilst patients with such mutations derive little, if any, benefit [83]. Where a single biomarker has been identified, several trial designs can be employed [84], including (i) biomarker-enrichment design, which involves only patients testing positive for the biomarker. This design is more appropriate when there is preliminary evidence that patients testing positive for the biomarker will likely benefit from the treatment; (ii) biomarker-stratified design involves first testing patients for the biomarker and then separately randomising patients who test positive and those who test negative. This design is more appropriate when there is no preliminary evidence to strongly favour a positive or negative biomarker. The medical literature in oncology provides good examples of biomarker-driven studies [85]. No similar trial strategies have been adopted in RA yet. Paradoxically, despite anticitrullinated protein antibodies (ACPA) are acknowledged as one of the strongest prognostic factors of worst disease outcomes [86], no clinical studies have tailored RA treatment based on a positive ACPA-test. However, evidence from the PROMPT study seems to suggest that early MTX treatment in patients with undifferentiated arthritis could significantly delay progression to RA specifically in ACPA-positive patients [87], confirming the possibility of identifying subgroups of patients with treatment benefit at least in the earliest phases of the disease [88]. The field of biomarker discovery is moving fast in RA, fuelled by the implementation of systems biology and omic technologies. The increased awareness of the systemic and multidistrict nature of the disease have expanded the possibility to search for novel biomarkers in different diseased compartments [89–93]. Promising prognostic biomarkers (to be further tested for their predictive ability) are emerging in the peripheral circulation [94–97], and the accessibility of the synovial tissue through minimally invasive techniques [98] allows more extensive studies aimed at investigating the clinical and prognostic significance of different pathological features [99, 100].

## 4. Head-to-Head Trials

Undoubtedly, the use of a placebo control in RCTs offers several advantages. Inclusion of placebo increases the efficiency of a trial, as statistical significance can be achieved with the smallest number of participants. Secondly, the results of a placebo-controlled trial are usually unequivocal, with clear evidence of whether the experimental drug being tested is efficacious or not. There are few ethical concerns in using placebo-controlled trials when no therapy of proven effectiveness exists. In contrast, when standard therapy does exist, controlling for placebo raises not only ethical but also practical issues, in that the usefulness of the results is ambiguous.

Despite the fact the regulatory agencies recommend that placebo should not be continued for more that 3–6 months in RA trials [101], Estellat and Ravaud [102], through a revision of all RCTs of biological DMARDs ended after 2002, highlighted that 6,518 RA patients enrolled in control arms were continuing their previously ineffective treatment for more than 6 months. As such, a significant revision in the requirements for the investigation and approval of new drugs for the treatment of RA is needed. An International Committee has recently proposed that placebo can be acceptable for no more than 3 months and new biological DMARDs should be tested against an active comparator [103]. In light of their tightest confidence intervals for efficacy, TNF-inhibitors (+ MTX) should be the comparator of choice [103].

Active control or comparative or head-to-head trials refer to all studies in which the control arm is an active one. Based on the scientific hypothesis behind the trial, comparative trials may be classified as superiority, equivalence, or noninferiority trials [104, 105].

In a superiority trial, the aim is to show that a new treatment is better than standard therapy. The null hypothesis is that the difference between the means of the two groups is zero or negative (i.e., favouring the standard treatment) versus the one-tailed alternative hypothesis that the new drug is better. The desirable difference in treatment effects should be decided on clinical grounds, considering the specific features of the disease, the known efficacy of the control therapy, and what may reasonably be expected from the new therapy. The rationale for a one-sided test of significance is that investigators are not interested in results that show that the new drug is equal to or inferior than the standard. However, a shortcoming of one-tailed tests is that significant results in the opposite direction must be dismissed as chance findings, despite having the potential of being clinically meaningful. Superiority trials are almost never seen in active-control trials because of the high risks of failure intrinsic in their design and the required sample size, which can be unachievable in certain conditions. Indeed, the smallest the difference between the standard and the experimental drug is expected, the largest will be the population required.

Equivalence trials test whether the effects of two drugs are the same within prespecified limits. As it is fundamentally impossible to prove that two treatments have exactly equivalent effects, “clinical equivalence intervals” must be determined. The null hypothesis is that the difference between treatments falls outside the interval versus the alternative hypothesis that the differences lie within the equivalence interval. Equivalence trials are based on two-sided tests, which increase the sample size and the cost of the study. Furthermore, equivalence margins are often far too large to be clinically meaningful and a claim of equivalence may be misleading if a trial has not been conducted to an appropriately high standard. Equivalence trials are often run when biosimilars are entering the market.

Noninferiority trials test whether the effect of a new treatment is not worse than that of an active control by more than a prespecified margin. Again, an interval of noninferiority must be determined. The null hypothesis is that the new drug is worse than the standard one by at least some amount, against the alternative hypothesis that the superiority of the standard drug does not exceed this interval. However, since noninferiority trials do not include a true negative control group, results of these trials are viewed with caution and are not generally accepted as being as strong as those from a superiority trial. Noninferiority trials are carried out when (1) a placebo-controlled trial is not ethically feasible and (2) the treatment under test is not expected to be better that the standard or reference intervention in terms of efficacy but is supposedly better regarding other secondary endpoints, safety, costs, compliance, or convenience.

## 5. Head-to-Head Randomised Controlled Trials in Rheumatoid Arthritis: State of the Art

### 5.1. Synthetic DMARD versus Synthetic DMARD

The comparison between two different synthetic DMARD monotherapies for RA was reported in 14 RCTs, all having MTX as basis for comparison (Table 2).

#### 5.1.1. Auranofin (AUR)

In a 36-week RCT randomising 281 patients with active RA to MTX or AUR, the clinical response (swollen joint count [SJC] and tender joint count [TJC]) with MTX occurred earlier and was consistently greater (*P* < 0.01) than the one with AUR. Adverse reactions were reported more frequently in the AUR group [106]. However, in a subsequent RCT involving 335 patients and comparing MTX, AUR, and the combination of both, no statistically significant differences were found among the treatment groups in terms of clinical response and safety profiles, even if patients taking AUR alone had a slower onset of response than those taking MTX alone [107].

#### 5.1.2. Azathioprine (AZA)

AZA was directly compared with MTX in two RCTs with similar results. In the first one, 64 patients were randomly assigned to receive either AZA (100 mg daily) or oral MTX (7.5 mg weekly): both clinical responses (SJC, erythrocyte sedimentation rate [ESR], C-reactive protein level [CRP], and DAS) at 24 and 48 weeks checkpoints [108] and radiographic progression [109] were significantly better in MTX treated group, accompanied by a lower rate of serious adverse reactions. In the second study [110], 209 enrolled patients were randomised to receive escalating doses of MTX (5–15 mg/week), AZA (50–150 mg/day), or the combination of both. The proportion of responders was significantly higher in MTX treated group compared with AZA (45% versus 26%, resp.) and a trend toward decreased radiologic progression was seen only in MTX-treated patients.

#### 5.1.3. Cyclosporine A (CSA)

Two RCTs compared CSA with MTX with different findings. Drosos et al. [111] demonstrated a similar clinical response (SJC, TJC, ESR, and CRP) and radiographic progression in two groups of early RA patients (disease duration < 3 years) randomly assigned to receive oral CSA (3 mg/kg/day) or oral MTX (0.15 mg/kg/week). More recently, in an open RCT randomising 126 patients to MTX, CSA, or SSZ, American College of Rheumatology (ACR) 50 responses were significantly higher in MTX treated patients compared with CSA (57% versus 31%, resp.; *P* = 0.002) [112]. In both the studies, the proportion of adverse events (AEs) was similar in MTX and CSA treated groups.

#### 5.1.4. Intramuscular Gold (Gold Sodium Thiomalate [GST])

GST showed a similar clinical response (ESR, CRP, Ritchie Articular Index, and pain score) and a significantly higher proportion of withdrawals for toxicity (43% GST versus 19% MTX, *P* = 0.0026) compared to MTX in a 48-week head-to-head RCT [113]. It should be noticed that in this study MTX was used at relatively lower doses (median dose: 10 mg/weekly) than the currently recommended optimal doses. In a second double-blind RCT evaluating damage progression, 174 patients were assigned to receive weekly intramuscular injections of either 15 mg MTX or 50 mg GST for 3 years. No statistically significant differences in clinical efficacy and radiographic progression between the 2 treatment groups at all follow-up points were found [114].

#### 5.1.5. Leflunomide (LEF)

As part of the clinical development program for LEF provided by the Leflunomide Rheumatoid Arthritis Investigators Groups, patients were enrolled in 2 RCTs comparing LEF with SSZ and MTX, respectively. Since the first one [115] was not powered to show equivalence between the active treatments but only to indirectly compare LEF and SSZ, the study has not been included in the current review. In the second one, 482 patients were randomly assigned to receive LEF (100 mg daily on days 1–3, then 20 mg daily), placebo, or MTX (7.5 mg weekly, titrated to 15 mg weekly over 7 weeks ). No statistically significant differences were found in the comparison of LEF and MTX treated patients regarding ACR20 response at 1- (52% versus 46%, resp.) [116] and 2-years follow-up (79% versus 67%, resp.) [117]. Moreover, radiographic progression at 1- and 2-year evaluations [116, 117] and improvements in physical function and health related quality of life at 2 years [118] were similar in the 2 treatment groups.

A direct comparison between LEF and MTX was performed in another RCT including 999 RA subjects randomised to LEF (loading dose 100 mg/day for 3 days, maintenance dose 20 mg/day) or MTX (10–15 mg/week) for 52 weeks [119]. After 1 year, improvements seen with MTX were significantly greater than those with LEF in terms of ACR20 response (64.8 versus 50.5%; *P* < 0.0001) and mean change from baseline of TJC (−9.7 versus −8.3; *P* = 0.006), SJC (−9.0 versus −6.8; *P* = 0.0001), physician global assessment (−1.2 versus −0.9; *P* < 0.001). Radiographic progression was similar with both treatment protocols at 1 year, whereas a significant difference in mean change from baseline of Larsen score in favour of MTX was found at 2 years. The proportion of AEs leading to withdrawal at 2 years (MTX 21%, LEF 27%) and the overall frequency of serious AEs (MTX 8%, LEF 7%) were comparable.

Besides, in a third comparative trial including 504 RA patients evaluated during a 24-week follow-up period, LFN was found as effective but safer than MTX [120]. In particular, 62% patients in the LFN group met the ACR20 criteria versus 60% in the MTX one, but the incidences of AEs were significantly lower in LFN than in MTX treated patients (16.8% versus 28.1%; *P* = 0.002).

#### 5.1.6. Sulfasalazine (SSZ)

A direct comparison of SSZ with MTX was performed in 3 RCTs [121–123], each designed by the randomisation of study population (105, 205, and 165 patients, resp.) into 3 treatment arms (SSZ alone [2000 to maximum 3000 mg daily], MTX alone [7.5 to maximum 25 mg weekly], and the combination of two). In all these studies, no significant differences emerged in the 1-year head-to-head comparison between SSZ and MTX in terms of clinical efficacy (measured as DAS), radiographic progression (total sharp score), and frequency of AEs.

On the contrary, in the previously mentioned study by Ferraccioli et al. [112] directly comparing MTX, CSA, and SSZ, the proportion of patients achieving ACR50 response at 12 months was significantly higher in MTX than in SSZ treated group (57% versus 33%, *P* < 0.01), with a similar safety profile.

### 5.2. Synthetic DMARD versus Biological DMARD

The vast majority of biotherapy related RCTs are designed to compare MTX monotherapy with the combination of MTX and a biological drug. Thus, only 4 RCTs provided data on the direct comparison between a synthetic and a biologic DMARD monotherapy: the ERA study and the TEMPO trial evaluated ETN, the PREMIER study ADA, and the AMBITION trial TCZ, all head-to-head compared against MTX (Table 2).

The ERA study is a 24-month RCT with both clinical and radiographic primary endpoints [14, 124]. In the study, 632 MTX-naïve early RA patients were randomised to receive either twice weekly subcutaneous ETN (10 or 25 mg) or weekly escalating doses of oral MTX (7.5–20 mg/week). The patients in the group assigned to the higher ETN dose had significantly greater areas under the curve for the numeric index of the ACR response [ACR-N AUC] at 3, 6, 9, and 12 months than did the patients in the MTX group (*P* < 0.05). However, no differences in the proportion of patients achieving ACR20 (72% versus 65%; *P* = 0.16), 50, and 70 responses at 12 months were found in the comparison of ETN and MTX treated groups. The mean increase in the erosion score was significantly lower in the 25-mg ETN group than in the MTX group at both 6-month (0.30 versus 0.68; *P* = 0.001) and 12-month (0.47 versus 1.03; *P* = 0.002) evaluations, as well as the mean total modified sharp score (mTSS) increases at 6 months (0.57 versus 1.06; *P* = 0.001), but not at 12 months (1.00 versus 1.59; *P* = 0.11) [14]. At 24 months, significantly more patients in the 25-mg ETN group than in the MTX group achieved an ACR20 response (72% versus 59%; *P* = 0.005) and the mean changes in mTSS and erosion score in the 25-mg ETN treated patients (1.3 and 0.66 units, resp.) were significantly lower than those in the MTX group (3.2 and 1.86 units, resp.; *P* < 0.001) [124]. The safety outcomes through the entire 2-year follow-up period were comparable between the two treatment drugs in terms of both infectious and noninfectious events, with the only exception of injection site reactions (ETN 25 mg 39% versus MTX 9%; *P* < 0.05), nausea (ETN 25 mg 14% versus MTX 31%; *P* < 0.05), alopecia (ETN 25 mg 6% versus MTX 12%; *P* < 0.05), and mouth ulcers (ETN 25 mg 5% versus MTX 17%; *P* < 0.05).

The AMBITION trial [125] is a 6-month RCT including 673 patients with active RA for whom previous treatment with MTX had not failed. Study population was randomly assigned either TCZ 8 mg/kg every 4 weeks, or MTX (starting at 7.5 mg/weekly and titrated to 20 mg/weekly within 8 weeks), with the proportion of patients achieving ACR20 response at week 24 as the primary endpoint. The intention-to-treat analysis demonstrated that TCZ monotherapy was better than MTX with higher ACR20 (69.9% versus 52.5%; *P* < 0.001), ACR50 (44.1% versus 33.5%; *P* = 0.002), and ACR70 (28% versus 15.1%; *P* < 0.001) responses. Moreover, TCZ patients were five times more likely to achieve DAS28 remission (odds ratio versus MTX: 5.83; 95% confidence interval [CI] 3.27 to 10.40), and approximately four times more likely to achieve at least a moderate EULAR response (odds ratio versus MTX: 4.24, 95% CI 2.92 to 6.14). No significant differences between TCZ and MTX were found in the incidence of AEs (79.9% versus 77.5%; *P* = 0.484), serious AEs (3.8% versus 2.8%, *P* = 0.50), and serious infections (1.4% versus 0.7%). Furthermore, TCZ treated patients showed a higher incidence of reversible grade-3 neutropenia (3.1% TCZ versus 0.4% MTX) and increased total cholesterol ≥240 mg/dL (13.2% TCZ versus 0.4% MTX), and a lower incidence of alanine aminotransferase elevations >3x–<5x upper limit of normal (1.0% TCZ versus 2.5% MTX).

In the TEMPO trial [27] 686 RA patients were randomised to receive oral MTX (up to 20 mg/week), ETN (25 mg twice a week), or the combination of both, with clinical (24 weeks ACR-N AUC) and radiological (52 weeks mTSS change from baseline) primary endpoints. The combination therapy was significantly better than ETN and MTX monotherapies in reduction of disease activity, improvement of functional disability, and retardation of radiographic progression.

Focusing on direct comparison between ETN and MTX groups, no statistically significant differences emerged in terms of 1-year clinical response (ACR20 76% versus 75%, ACR50 48% versus 43%, and ACR70 24% versus 19%, resp.), whereas 1-year damage worsening was significantly lower in the ETN group compared with MTX (proportion of patient without progression 68% versus 57%, resp.; *P* = 0.02. Mean mTSS change from baseline 0.52 versus 2.80, resp.; *P* = 0.04). This finding was confirmed by 2-years follow-up data, showing a significantly lower mean change from baseline in mTTS in patients receiving ETN compared with those receiving MTX (1.10 versus 3.34, resp.; *P* = 0.05) [123]. The proportion of patients reporting AEs (ETN 86% versus MTX 81%) or serious infections (ETN 4% versus MTX 4%) was comparable between ETN and MTX groups.

As well as TEMPO trial, PREMIER study [12] was designed by randomising 799 early MTX-naïve RA patients into 3 treatment arms (oral MTX 20 mg/weekly, ADA 40 mg/every other week, or the combination of both). Coprimary endpoints at year 1 were ACR50 improvement and mean change from baseline in the mTTS. Similarly to what previously reported in the TEMPO trial, combination therapy was superior to both ADA and MTX monotherapies in all clinical and radiographic outcomes measured, whereas the proportion of 12- and 24-month ACR20 (54% versus 63% and 49% versus 56%, resp.), ACR50 (41% versus 46% and 37% versus 43%, resp.), and ACR70 (26% versus 28% and 28% versus 28%, resp.) responses were comparable between ADA and MTX groups. Otherwise, damage progression at both 1- and 2-year evaluations was significantly lower in ADA treated patients directly compared with MTX treated ones (mean change from baseline in mTTS 3.0 versus 5.7 and 5.5 versus 10.4, resp.; *P* < 0.001). The incidence of serious AEs and serious infections (21.1 versus 15.9 and 0.7 versus 1.6 per 100 patient years, resp.) was similar in both monotherapy groups.

### 5.3. Biological DMARDs versus Biological DMARDs

The head-to-head comparison between 2 different biological agents is available only in 2 RCTs, the ADACTA trial (comparing TCZ and ADA monotherapies) and the AMPLE trial (comparing ABT and ADA, both on top of MTX) (Table 2). The ATTEST study [20] and the ORAL STANDARD study [126] were excluded since they were designed with the statistical power for comparing biologic drugs against a common placebo group only but not against each other.

ADACTA is the first head-to-head superiority RCT comparing TCZ (8 mg/kg every 4 weeks) and ADA (40 mg every other week) monotherapy in a study population of MTX-insufficient responder RA patients (*n* = 335) [127]. TCZ monotherapy was superior to ADA monotherapy at 6 months according to all main efficacy endpoints: EULAR remission (39.9% versus 10.5%; *P* < 0.0001), EULAR low disease activity (51.5% versus 19.8%; *P* < 0.0001), ACR20 (65% versus 49.4%; *P* = 0.003), ACR50 (47.2% versus 27.8%; *P* = 0.0002), ACR70 (32.5% versus 17.9%; *P* = 0.002), and CDAI remission (17.2% versus 9.3%; *P* = 0.03). The kinetics of clinical response in the two groups were comparable through the entire follow-up period. The adverse event profiles of TCZ and ADA were similar and consistent with previous findings. In particular, no significant differences were found between the 2 treatment groups in the 6-month rate of overall (82% versus 83%) and serious (12% versus 10%) AEs, and overall (70% versus 65%) and serious (4% versus 4%) infections, as well as in the frequency of transaminase elevation ≤2.5x upper limit of normal (32% versus 25%). Otherwise, some differences in the effect of the two drugs on the neutrophil count have been registered, with a proportion of patients experiencing grade-2 neutropenia (<1500–1000x mm^3^) with TCZ higher than with ADA (13% versus 4%), even if cases of severe neutropenia were very rare in both the treatment groups.

The AMPLE trial provided a noninferiority comparison of subcutaneous ABT and ADA, both administrated in combination with MTX, in a study population of 646 RA patients through a 2-year follow-up period [128, 129]. The clinical efficacy of ABT and ADA is comparable according to 1 and 2 years ACR20 (64.8% versus 63.8% and 60.1% versus 59.7%, resp.), ACR50 (46.2% versus 46% and 46.6% versus 44.7%, resp.), and ACR70 improvements (29.2% versus 26.2% and 31.1% versus 29.3, resp.), with similar kinetics of response throughout the entire 2-year follow-up period. Similarly, no significant differences emerged in the comparison of radiographic progression in ABT and ADA treated patients, with more than 80% nonprogressor patients in both the groups. Finally, 2-year safety outcomes are balanced, but with some notable differences in the incidence of AEs (10.1% versus 9.1%), serious AEs (1.6% versus 4.9%), and serious infections (0 versus 2.7%) and injection site reactions (4.1% versus 10.4%), all in favour of ABT compared with ADA.

## 6. Conclusions

The arsenal of therapeutic options for RA is vast, but knowledge on the optimal use of different drugs in individual patients in typical day-to-day practice remains poor. Over a period of more than 25 years, only 20 head-to-head RCTs comparing two different DMARDs have been performed, providing some preliminary but encouraging suggestion on how to deal with the complexity of the available therapeutic armamentarium. Key messages emerging from direct comparisons are as follows.

- MTX overall risk/benefit ratio is the most favourable compared with other synthetic DMARDs, confirming its use as first line therapy and LEF or SSZ as an alternative treatment in newly diagnosed RA, as suggested by international guidelines [53].
- TCZ is the only biologic DMARD with a demonstrated clinical superiority compared to MTX. ETN and ADA have been shown to be able only to slow damage progression better than MTX, without significant differences in clinical response.
- TCZ monotherapy is superior to ADA monotherapy, with a similar safety profile.
- Clinical efficacy, damage progression, and kinetics of response of sc ABT and ADA are comparable. Safety profiles are quite similar, slightly in favour of ABT.

It is hoped that future years will witness a radical shift in the way medical research is conceived and performed in RA. More direct comparisons and innovative trial designs will help achieving the final goal of treating the right patient at the right time with the right drug.

## Figures and Tables

**Table 1:** Randomised controlled trials of biological disease-modifying anti-rheumatic drugs that have supported regulatory labeling.

	Trial	Study drug	Duration	Primary endpoints
MTX-na*ї*ve				
abatacept*	AGREE [13]	ABT + MTX versus MTX	12 months	DAS28-CRP < 2.6 at 12 months xRay progression at 12 months
adalimumab	PREMIER [14]	ADA + MTX versus MTX versus ADA	24 months	ACR50 at 12 months xRay progression at 12 months
	OPTIMA [15]	ADA + MTX versus PL + MTX	6 months	DAS28-CRP < 3.2 at 78 wks no xRay progression at 78 wks
certolizumab*	—			
etanercept	ERA [16]	ETN versus MTX	12 months	overall ACR response at 6 months xRay progression at 12 months
	COMET [17]	ETN + MTX versus MTX	24 months	DAS28 < 2.6 at 12 months xRay progression at 12 months
golimumab	GO-BEFORE [18]	GOL + MTX versus PL + GOL versus PL + MTX	6 months	ACR50 at 6 months
infliximab	ASPIRE [19]	IFL + MTX versus PL + MTX	12 months	overall ACR response at 12 months xRay progression at 12 months
rituximab**	IMAGE [20]	RTX + MTX versus MTX	12 months	xRay progression at 12 months
tocilizumab**	—			
MTX/DMARD-IR				
abatacept	AIM [21]	ABT + MTX versus PL + MTX	12 months	ACR20 at 6 months HAQ-DI at 12 months xRay progression at 12 months
	ATTEST [22]	ABT + MTX versus PL + MTX versus PL + INF	12 months	DAS28 at 6 months
adalimumab	ARMADA [23]	ADA + MTX versus PL + MTX	6 months	ACR20
	DE019 [24]	ADA + MTX versus PL + MTX	12 months	ACR20 at 6 months HAQ-DI at 12 months xRay progression at 12 months
certolizumab	RAPID-1 [25]	CER + MTX versus PL + MTX	12 months	ACR20 at 6 months xRay progression at 12 months
	RAPID-2 [26]	CER + MTX versus PL + MTX	6 months	ACR20 at 6 months
	FAST4WARD [27]	CER versus PL	6 months	ACR20 at 6 months
etanercept	Weinblatt [28]	ETN + MTX versus PL + MTX	6 months	ACR20 at 6 months
	TEMPO [29]	ETN versus MTX versus ETN + MTX	12 months	overall ACR response at 6 months xRay progression at 12 months
	ADORE [30]	ETN + MTX versus ETN	4 months	improvement of > 1.2 units in DAS28 at 4 months
golimumab	GO-FORWARD [31]	GOL + MTX versus PL + GOL versus PL + MTX	6 months	ACR20 at 14 weeks HAQ-DI at 6 months
infliximab	ATTRACT [32]	IFL + MTX versus PL + MTX	30 weeks	ACR20 at 30 weeks
rituximab***	DANCER [33]	RTX + MTX versus PL + MTX	6 months	ACR20 at 6 months
	SERENE [34]	RTX + MTX versus PL + MTX	12 months	ACR20 at 6 months
	MIRROR [35]	RTX + MTX versus PL + MTX	12 months	ACR20 at 12 months
tocilizumab	OPTION [36]	TCZ + MTX versus PL + MTX	6 months	ACR20 at 6 months
	LITHE [37]	TCZ + MTX versus PL + MTX	24 months	HAQ-DI at 12 months xRay progression at 12 months
	TOWARD [38]	TCZ + DMARD versus PL + DMARD	6 months	ACR20 at 6 months
	STREAM [39]	TCZ versus PL	3 months	ACR20 at 3 months
	ROSE [40]	TCZ + DMARD versus PL + DMARD	6 months	ACR20 at 6 months
Anti-TNF IR				
abatacept	ATTAIN [41]	ABT + DMARD versus PL + DMARD	6 months	ACR20 at 6 months HAQ-DI at 6 months
adalimumab	—			
certolizumab	—			
etanercept	—			
golimumab	GO-AFTER [42]	GOL + DMARDs versus PL + DMARDs	6 months	ACR20 at 14 weeks
infliximab	OPPOSITE [43]	IFL + MTX versus ETN + MTX	n.d.	none
rituximab	REFLEX [44]	RTX + MTX versus PL + MTX	24 months	ACR20 at 6 months
tocilizumab	RADIATE [45]	TCZ + MTX versus PL + MTX	6 months	ACR20 at 6 months

*approved by FDA, not approved by EMA in MTX-na*ї*ve patients.

**not approved by neither FDA nor by EMA in MTX-na*ї*ve patients.

***not approved by neither FDA nor by EMA in MTX-IR patients.

ABT: abatacept; ACR: American College of Rheumatology; ADA: adalimumab; CER: certolizumab; CRP: C-reactive protein; DAS: disease activity score; DMARD: disease modifying anti-rheumatic drug; ETN: etanercept; GOL: golimumab; HAQ-DI: Health Assessment Questionnaire Disability Index; IFL: infliximab; IR: irresponsive; MTX: methotrexate; PL: placebo; RTX: rituximab; TCZ: tocilizumab; TNF: tumor necrosis factor.

**Table 2:** Summary of head-to-head trials in rheumatoid arthritis.

Reference	Study design	Drugs	Follow-up	Number of patients	Primary endpoint	Results
sDMARDs versus sDMARDs						
Weinblatt et al., 1990 [106]	Randomized double-blind controlled	AUR versus MTX	36 wks	138 versus 142	TJC, SJC, PhGA, PtGA	MTX is more effective and better tolerated than AUR
Williams et al., 1992 [107]	Randomized double-blind controlled	AUR versus MTX versus AUR + MTX	48 wks	115 versus 114 versus 106	TJC, SJC, PhGA, PtGA	No differences
Jeurissen et al., 1991 [108, 109]	Randomized double-blind controlled	AZA versus MTX	48 wks	33 versus 31	Ritchie index, TJC, SJC, VAS pain, PtGA	MTX is more efficacious and more rapid than AZA
Willkens et al., 1995 [110]	Randomized double-blind controlled	AZA versus MTX versus AZA + MTX	48 wks	73 versus 67 versus 69	TJC, SJC, PhGA, PtGA, HAQ, mTSS	MTX is more efficacious than AZA. Trend toward decrease radiographic progression only in MTX
Drosos et al., 1998 [111]	Randomized open labeled trial	CSA versus MTX	104 wks	52 versus 51	TJC, SJC, VAS pain, Larsen score	No differences in efficacy and radiographic progression
Ferraccioli et al., 2002 [112]	Open randomized controlled	SSZ versus MTX versus CsA	24 wks	42 versus 42 versus 42	ACR50	MTX is more efficacious than CSA and SSZ
Hamilton et al., 2001 [113]	Randomized open labeled trial	GST versus MTX	48 wks	72 versus 69	Paulus response criteria	GST and low dose MTX showed equivalent efficacy, but toxicity was more common in GST
Rau et al., 2002 [114]	Randomized double-blind controlled	GST versus MTX	156 wks	87 versus 87	Ratingen score	No differences in clinical efficacy and radiographic progression
Strand et al., 1999 [116]	Randomized double-blind controlled	LFN versus Placebo versus MTX	52 wks	182 versus 118 versus 182	ACR20	No differences in the efficacy of MTX versu LFN
Emery et al., 2000 [119]	Randomized double-blind controlled	LFN versus MTX	52 wks	501 versus 498	TJC, SJC, PhGA, PtGA	MTX is more efficacious than LEF, with low 2-yrs radiographic progression
Bao et al., 2003 [120]	Open randomized controlled	LFN versus MTX	24 wks	291 versus 213	ACR20	LFN is as effective but safer than MTX
Haagsma et al., 1997 [121]	Randomized double-blind controlled	SSZ versus MTX versus SSZ + MTX	52 wks	34 versus 35 versus 36	DAS	No differences in efficacy and radiographic progression between MTX and SSZ
Dougados et al., 1999 [122]	Randomized double-blind controlled	SSZ versus MTX versus SSZ + MTX	52 wks	68 versus 69 versus 68	DAS	No differences in efficacy and radiographic progression between MTX and SSZ
Capell et al., 2007 [123]	Randomized double-blind controlled	SSZ versus MTX versus SSZ + MTX	52 wks	55 versus 54 versus 56	DAS	No differences in efficacy and radiographic progression between MTX and SSZ
sDMARDs versus bDMARDs						
Bathon et al., 2000 [16]	Randomized double-blind controlled	ETN 25 mg versus ETN 10 mg versus MTX	52 wks	207 versus 208 versus 217	ACR-N AUC (24 wks), mTSS (52 wks)	ETN had a more rapid rate of improvement than MTX
Jones et al., 2010 [125]	Randomized double-blind controlled	TCZ versus MTX	24 wks	288 versus 284	ACR20	TCZ monotherapy is more efficacious than MTX
Klareskog et al., 2004 [29]	Randomized double-blind controlled	ETN + MTX versus ETN Versus MTX	52 wks	231 versus 223 versus 228	ACR-N AUC (24 wks), mTSS (52 wks)	Combination therapy and ETN are more efficacious than MTX (combo > ETN).
Breedveld et al., 2006 [14]	Randomized double-blind controlled	ADA + MTX versus ADA versus MTX	104 wks	268 versus 274 versus 257	ACR50, mTSS	Combination therapy was superior to both mono-therapies. No differences between ADA and MTX.
bDMARDs versus bDMARDs						
Gabay et al., 2013 [127]	Randomized double-blind controlled	TCZ versus ADA	24 wks	163 versus 162	DAS28	TCZ is superior to ADA
Weinblatt et al., 2012 [128]	Randomized double-blind controlled	ABT versus ADA	52 wks	318 versus 328	ACR20, mTSS	ABT is noninferior to ADA

sDMARDs: synthetic disease modifying antirheumatic drugs; LFN: leflunomide; SSZ; sulfasalazine; TJC: tender joint count; SJC: swollen joint count; ACR: American College of Rheumatology; HAQ: Health Assessment Questionnaire; AZA: azathioprine; MTX: methotrexate; VAS: visual anagogic scale; GST: gold sodium thiomalate; AUR: auranofin; MRI: magnetic resonance imaging; LOCF: last observation carried forward; HRQOL: health related quality of life, SF-36: 36-item short form health survey, DAS: disease activity score; CsA: cyclosporine A; ETN: etanercept; ACR-N AUC: numeric index of the ACR response area under the curve; ADA: adalimumab; TCZ: tocilizumab; and ABT: abatacept.
